# Phagocytosis Is the Sole Arm of *Drosophila melanogaster* Known Host Defenses That Provides Some Protection Against Microsporidia Infection

**DOI:** 10.3389/fimmu.2022.858360

**Published:** 2022-04-13

**Authors:** Gaëtan Caravello, Adrien Franchet, Sebastian Niehus, Dominique Ferrandon

**Affiliations:** UPR9022, University of Strasbourg, Institut de Biologie Moléculaire et Cellulaire (IBMC), Modèles Insectes D’Immunité Innée (M3I) Unité Propre Recherche (UPR) 9022 du Centre National de la Recherche Scientifique (CNRS), Strasbourg, France

**Keywords:** *Tubulinosema ratisbonensis*, phagocytosis, *Drosophila*, intracellular parasite, immunity, mutant analysis

## Abstract

Microsporidia are obligate intracellular parasites able to infest specifically a large range of species, including insects. The knowledge about the biology of microsporidial infections remains confined to mostly descriptive studies, including molecular approaches such as transcriptomics or proteomics. Thus, functional data to understand insect host defenses are currently lacking. Here, we have undertaken a genetic analysis of known host defenses of the *Drosophila melanogaster* using an infection model whereby *Tubulinosema ratisbonensis* spores are directly injected in this insect. We find that phagocytosis does confer some protection in this infection model. In contrast, the systemic immune response, extracellular reactive oxygen species, thioester proteins, xenophagy, and intracellular antiviral response pathways do not appear to be involved in the resistance against this parasite. Unexpectedly, several genes such as *PGRP-LE* seem to promote this infection. The prophenol oxidases that mediate melanization have different functions; *PPO1* presents a phenotype similar to that of *PGRP-LE* whereas that of *PPO2* suggests a function in the resilience to infection. Similarly, *eiger* and *Unpaired3*, which encode two cytokines secreted by hemocytes display a resilience phenotype with a strong susceptibility to *T. ratisbonensis*.

## Introduction

*Microsporidia* are a group of more than 1,400 species of obligate intracellular parasites placed at the root of the fungal kingdom that are able to infect a large array of animal hosts, vertebrates and invertebrates (1). These pathogens are well adapted to their hosts, as attested by their reduced genomically-encoded metabolism and in some cases highly compacted genomes (2). Indeed, these parasites have lost numerous metabolic pathways, such as *de novo* synthesis pathways for amino acids, nucleotides; importantly, they lack mitochondria (2–5). *Microsporidia* infect their hosts by delivering the spore content directly or indirectly into the cytoplasm of the host cell through a polar tube (6). Next, the sporoplasm forms meronts in the host cell cytoplasm that multiply until filling the cell prior to differentiating into sporonts, sporoblasts, and then into mature spores. However, the signals triggering the eversion of the polar tube from the spore are generally scarcely known although several stimuli such as hydration or ultraviolet light have been described for some microsporidia species (7–9).

In humans, microsporidia essentially behave as opportunistic parasites. Human infections have increased at the turn of the century due to increased rates of immunodeficient AIDS patients, and microsporidia infections mostly affected the gut and/or the brain (10). Other microsporidia species like *Nosema cerenae* may contribute to honeybee colony collapse disorder alongside other factors (11). Indeed, it was reported that honeybees succumb even faster when also exposed to sub-lethal doses of pesticides, *e.g.*, the phenyl pyrazole fipronil (12). Some species are also inducing the formation of giant specialized structures called xenomas, syncytia resulting from the fusion of infected host cells that become parasite-producing factories (13, 14).

In vertebrates, both innate and adaptive immune responses have a role in controlling microsporidia infections. Innate immune cells such as, γδT cells, natural killer cells (NKs), macrophages and dendritic cells (DCs) are able to partially eliminate and/or present the spores to lymphocytes. However, the host requires the adaptive immune responses including cytototoxic T lymphocytes (CTL) and humoral immune responses to completely eliminate the parasite (15). Classically activated (M1) macrophages were shown to reduce *Encephalitozoon* infection (16–18) through a process that involves reactive nitrogen species as well as reactive oxygen species (ROS) (17, 19, 20). Macrophages can also secrete chemokines in response to microsporidia, which relies on the TLR2-NF-κB signaling pathway (21). Reports also highlight the important roles of IFN-γ and IL-12 in DCs response upon infection (22, 23), and the role of NKs (24). Moreover, several antimicrobial peptides were shown to have an effect on spore germination while reducing the infection of enterocytes (25). Finally, T-cell mediated immunity was shown to confer a critical protection against microsporidia (26).

In *Caenorhabditis elegans*, the parasites successfully invade epithelial cells, suggesting that defenses from these cells are not sufficient to control the infection (27). Nevertheless, ubiquitylation components, the proteasome, and autophagy have been shown to limit *Nematocida parisii* infection (28) while the p38 Mitogen-Associated Protein Kinases (MAPKs) and insulin/insulin-like growth factor (IGF) signaling pathways do not have roles in the resistance against microsporidia. More recently, a transcriptional immune/stress response called the intracellular pathogen response (IPR) has been shown to be triggered by microsporidia or viral infections as well as by proteotoxic stress and mutations in a purine nucleoside phosphorylase enzyme. These distinct activation pathways converge on the ZIP-1 transcription factor, which likely acts through the gene *pals-5* in the intestine (28–31). While these studies constitute major advances in our understanding of defenses against microsporidia in protostomes, the IPR may be specific to nematode worms. Indeed, most genes involved in the IPR do not appear to have homologues in the *D. melanogaster* genome.

Some host defenses such as the Toll pathway-mediated systemic immune response, phagocytosis, melanization or autophagy are believed to fight microsporidia infection in other invertebrates. *Nosema bombycis* is a pathogen of the silkworm *Bombyx mori* that contributed to “*pébrine*”, which caused important economic losses to the silkworm industry in the 19^th^ century. Transcriptomic studies revealed that potential host defenses including autophagy, oxidative stress, Toll, JAK-STAT and antimicrobial peptides (AMPs) are induced upon the infection while melanization was suppressed, suggesting that these pathways might be involved in the fight against these intracellular parasites in the silkworm (32–34). In *Aedes aegypti*, a study showed that antimicrobial defensins are upregulated upon *Vavraia culiculis* infection (35). Moreover, infected honeybees exhibited an increase in midgut oxidative stress (36–38). However, the parasites are able to suppress honeybee immunity as shown by the downregulation of genes coding for serine proteases, glucose dehydrogenase, lysozyme, GMC oxidoreductase, AMPs, dopa decarboxylase and catalases (36, 39). In *Drosophila*, one transcriptomic study showed that genes potentially involved in the host defenses, such as lysozyme and a scavenger receptor from the CD36 family coding genes, were induced upon ingestion of *Octosporea* (40). The biology of the intestinal *Octosporea* infection has not been described, thus limiting the interpretation of these data.

*Tubulinosema ratisbonensis* has been identified in a laboratory colony of *Drosophila melanogaster* (41). *In vitro*, the parasite is also able to infect insect and human cells (42). Previously, we described that *T. ratisbonensis* hijacks a specific metabolite playing a key role in the biosynthesis of triglycerides, phosphatidic acid, and thereby enhances its proliferation (43). In our infection model, we inject a controlled dose of spores directly into the fly hemolymph, perhaps inducing several immune pathways before intracellular infections of host tissues including the fat body occur. Few intracellular responses are known in *Drosophila* and have been essentially described for some bacterial and viral infections. Of note, adult *Drosophila* flies do not appear to be infected *per os* by *Tubulinosema* species, in contrast to larvae (44).

*Drosophila melanogaster* is often used as a model to study host-pathogen interactions in the framework of bacterial, viral, or fungal infections. Indeed, the fruit fly is able to fight most of infections through a variety of immune defenses including the systemic immune response (Toll, IMD, JAK-STAT & JNK pathways), the local immune response (AMP expression & ROS production by barrier epithelia) and the cellular immune response (phagocytosis, opsonization, encapsulation, coagulation and melanization) (45). In addition, some intracellular defenses have been documented *in vivo* such as Peptidoglycan Recognition Protein-LE (PGRP-LE)-mediated xenophagy of *Listeria monocytogenes* (46) and the Dicer2-Ago2 RNAi (47) as well as the Sting-Relish pathways as antiviral defenses (48–50). However, how *Drosophila* or insects reacts to microsporidia infection remains poorly explored (51). Furthermore, almost no infection models implicating a eukaryotic intracellular pathogen are described in this model insect. As the impact of microsporidia on economically important invertebrates (*e.g*., silkworm, shrimps) and broadly on insect populations (*e.g*., honeybees, mosquitoes) has been increasing over the years, using *Drosophila melanogaster* and its genetic tools and knowledge will be helpful to investigate insect host-defenses to the parasite. Indeed, a current limitation of the study of these defenses is that they rely essentially on descriptive transcriptomic and proteomic studies (51, 52) but are rarely followed up by experimental characterization. In *Drosophila*, it is easier to directly test the functional relevance of specific host defenses by genetic loss-of-function approaches. For instance, it is possible to silence gene expression in a specific tissue or cell-type by transgenic RNA interference (RNAi) (53). Alternatively, classical genetic mutants can be used when available and not affecting essential genes. We shall generically refer to RNAi or classical mutants simply as mutants. If a gene is specifically involved in resistance to *T. ratisbonensis* infection, the expectation is that the cognate mutant line will display a higher susceptibility to this infection in survival experiments and also an increased parasitic burden due to the heightened proliferation of the pathogen in immunodeficient flies. In contrast, if a gene is required in disease tolerance or resilience (54–56), homeostatic processes that help the host cope with damages inflicted during infection, its corresponding mutant will also display an increased sensitivity to the parasite but without any clear-cut impact on the microsporidial load (57). Alternatively, if the gene product is used by the parasite to enhance its infectivity, survival and microbial loads opposite to those of resistance mutants are expected.

Here, we functionally test using a genetic approach the known facets of the adult *Drosophila melanogaster* host defense to infections by its natural intracellular parasite *T. ratisbonensis* in a spore injection model. Unexpectedly, we found that most of the immune defenses including Toll, IMD, JAK-STAT, JNK, xenophagy, RNAi, STING, melanization & complement-related thioester-containing proteins (TEPs) are not required to control the parasite. The exception is phagocytosis that is effective to some degree against the parasite. We however did not identify receptors involved in the specific recognition of the parasite in *Drosophila*. Some signal transduction pathways yielded rather ambiguous phenotypes and we have observed in several instances an uncoupling between the survival phenotype and the microsporidial burden. Finally, some host defenses such as melanization are paradoxically required for the parasite to proliferate.

## Materials and Methods

### Parasite Culture

The microsporidia *T. ratisbonensis* was propagated and harvested as described (42, 58). The human lung fibroblast (MRC-5) cells used for this purpose (a gift from Thomas Baumert) were grown in DMEM + GlutaMAX (Gibco), supplemented with 10% (v/v) FCS and 1% (v/v) PenStrep (Invitrogen) in a tissue culture incubator under 5% CO2 at 37°C. The cell culture was intermittently tested for the presence of mycoplasma. The MRC-5 cell line was not authenticated and does not appear in the databases of commonly misidentified cell lines maintained by ICLAC and NCBI Biosample.

### Fly Strains

Fly lines were raised at 25°C with 60% humidity on a standard medium composed of 25 L of sterile water containing 1.2 kg cornmeal (Primeíal), 1.2 kg glucose (Tereos Syral), 1.5 kg yeast (Bio Springer), 90 g nipagin (VWR Chemicals) diluted into 350 ml ethanol (Sigma-Aldrich), and 120 g of agar-agar (Sobigel). Female flies were used in all experiments.

For experiments using mutant flies, *w^A5001^*, *w^1118^*, *yw*, or Canton S flies were used as wild-type controls as needed. *MyD88^c03881^*, *Tep3* and *Tep4* mutants were isogenized in the *w^A5001^* background (59). To silence gene expression ubiquitously or specifically in the fat body or in hemocytes, *Ubi-Gal4-Gal80^ts^*, *Yolk-Gal4*, or *Hml-Gal4-Gal80^ts^* virgin females were respectively crossed to males carrying relevant UAS-RNAi transgenes from the Vienna Drosophila RNAi Center (VDRC) or from the Transgenic RNAi Project (TRiP) at Harvard Medical School (Boston, MA, USA) (53, 60). For the VDRC RNAi flies, control *w^1118^* (no. 60000) were used for the GD construct and control *w^1118^* (no. 60200) were used for shRNA lines. For TRiP RNAi flies, the control flies were *mCherry VALIUM20* (no.35785). To check autophagy flux, we crossed *Ubi-Gal4-Gal80^ts^* virgins females with males carrying *UAS-GFP-mCherry-Atg8a*; the fusion of autophagosomes with lysosomes is quenching GFP fluorescence by the acidic hydrolases, resulting in red autolysosomes (61). Trans heterozygous *Atg7^d14^/Atg7^d77^* mutant flies were generated as described (62). To generate *hemoless* flies, virgin females *Hml-Gal4-UAS-GFP* were crossed with males carrying both *UAS-rpr* and *UAS-hid* transgenes. Crosses done with *Ubi-Gal4-Gal80^ts^ or Hml-Gal4-Gal80^ts^* were launched at 18°C and the progeny was harvested and kept at 29°C for 7 days before performing the experiment. Crosses done with *Yolk-Gal4* were launched at 25°C. For the generation of *hemoless* flies, crosses were launched and kept at 29°C during all developmental stages. Efficiency of hemocytes ablation was controlled by checking Hemocytes-GFP signal under the fluorescent microscope (**Figure S5A**). The effectiveness of autophagy inhibition was checked by performing starvation experiments (in mutant or knockdown flies). We decided to work mostly on Atg7 lines as they were the ones displaying the strongest phenotype in starvation experiments. Using the proper wild-type control in all experiments was important as the fly genetic background impacts the fly resistance to the spores. All fly lines used in this study and their origins are described in **Table 1**.

**Table 1:** Summary of fly lines used in this study.

Fly strain	Origin	Stock Number	Type
*w^A5001^*		N/A	wild-type
Drosdel *w^1118^* iso	Gift from Bruno Lemaitre	N/A	wild-type
*w^1118^* (*dSTING^-/-^* control)	Gift from Akira Goto	N/A	wild-type
*yw*	Gift from Akira Goto	N/A	wild-type
*Canton S*		N/A	wild-type
*MyD88^c03881^*	(63)	N/A	mutant
*kenny^-/-^*	(64)	N/A	mutant
*ΔAMPs*	(65)	N/A	mutant
*PGRP-LE^112^*	Bloomington Drosophila stock center	BDSC_33055	mutant
*Atg7^d14^*	(62)	N/A	mutant
*Atg7^d77^*	(62)	N/A	mutant
*CG5335^d30^*	(62)	N/A	mutant
*Dicer-2^null^*	(66)	N/A	mutant
*Dicer-2^Rescue^*	(66)	N/A	mutant
*dSTING^-/-^*	(48)	N/A	mutant
*PPO1^Δ^*	(67)	N/A	mutant
*PPO2^Δ^*	(67)	N/A	mutant
*PPO1^Δ^,2^Δ^*	(67)	N/A	mutant
*eater^-/-^*	(68)	N/A	mutant
*drpr^HP37013^*	Bloomington Drosophila stock center	BDSC_22010	mutant
*NimA^MI11280^*	Bloomington Drosophila stock center	BDSC_56414	mutant
*Tep1^-/-^*	Gift from Bruno Lemaitre	N/A	mutant
*Tep2^-/-^*	(69)	N/A	mutant
*Tep3^-/-^*	(69)	N/A	mutant
*Tep4^-/-^*	(69)	N/A	mutant
*Tepq ^Δ^*	(70)	N/A	mutant
*NOS^Δ15^*	(71)	N/A	mutant
*Ubi-Gal4-Gal80^ts^*	This laboratory	N/A	driver
*Yolk-Gal4*	This laboratory	N/A	driver
*Hml-Gal4-UAS-GFP*	Bloomington Drosophila stock center	BDSC_30140	driver
*Hml-Gal4-Gal80^ts^*	This laboratory	N/A	driver
*Dipt-LacZ*	(72)	N/A	reporter
*Drosomycin-GFP*	(73)	N/A	reporter
*UAS-rpr;UAS-hid*	Gift from Shigeo Hayashi	N/A	overexpression
*UAS-GFP*	Bloomington Drosophila stock center	BDSC_1522	overexpression
*UAS-nls-mCherry*	Bloomington Drosophila stock center	BDSC_38424	overexpression
*UAS-GFP-mCherry-Atg8a*	Bloomington Drosophila stock center	BDSC_37749	overexpression
*UAS-mCherry TRiP control*	Bloomington Drosophila stock center	BDSC_35785	RNAi-TRiP
*GD w^1118^ control*	Vienna Drosophila Resource Center	VDRC: 60000	RNAi-GD
*TK w^1118^ control*	Vienna Drosophila Resource Center	VDRC: 60200	RNAi-TK
*UAS-basket RNAi*	Bloomington Drosophila stock center	BDSC_57035	RNAi-TRiP
*UAS-ask1 RNAi*	Bloomington Drosophila stock center	BDSC_35331	RNAi-TRiP
*UAS-p38b RNAi*	Bloomington Drosophila stock center	BDSC_35252	RNAi-TRiP
*UAS-PGRP-LE RNAi*	Vienna Drosophila Resource Center	VDRC: 23664	RNAi-GD
*UAS-Atg5 RNAi*	Bloomington Drosophila stock center	BDSC_34899	RNAi-TRiP
*UAS-Atg7 RNAi*	Bloomington Drosophila stock center	BDSC_34369	RNAi-TRiP
*UAS-Atg8a RNAi*	Bloomington Drosophila stock center	BDSC_34340	RNAi-TRiP
*UAS-Duox RNAi*	Bloomington Drosophila stock center	BDSC_33085	RNAi-TK
*UAS-NOX RNAi*	Gift from Sino-French Hoffmann Institute, China	N/A	RNAi
*UAS-nimB1 RNAi*	Bloomington Drosophila stock center	BDSC_55937	RNAi-TRiP
*UAS-nimB2 RNAi*	Bloomington Drosophila stock center	BDSC_65098	RNAi-TRiP
*UAS-nimB2 RNAi*	Bloomington Drosophila stock center	BDSC_62289	RNAi-TRiP
*UAS-nimB4 RNAi*	Bloomington Drosophila stock center	BDSC_55963	RNAi-TRiP
*UAS-nimB4 RNAi*	Bloomington Drosophila stock center	BDSC_62890	RNAi-TRiP
*UAS-nimB5 RNAi*	Bloomington Drosophila stock center	BDSC_51162	RNAi-TRiP
*UAS-nimC1 RNAi*	Bloomington Drosophila stock center	BDSC_25787	RNAi-TRiP
*UAS-crq RNAi*	Bloomington Drosophila stock center	BDSC_40831	RNAi-TRiP
*UAS-pes RNAi*	Bloomington Drosophila stock center	BDSC_50612	RNAi-TRiP
*UAS-pvf2 RNAi*	Bloomington Drosophila stock center	BDSC_61955	RNAi-TRiP
*UAS-eiger RNAi*	Bloomington Drosophila stock center	BDSC_55276	RNAi-TRiP
*UAS-upd3 RNAi*	Bloomington Drosophila stock center	BDSC_28675	RNAi-TRiP

N/A, no answer, because there are no stock number for these lines.

### Microsporidia Infection

For microsporidia infection, spores were stored in PBS at 4°C. Microsporidia spores were injected into the thorax, precisely into the mesopleuron on adult flies at a concentration of 2,000 spores (unless indicated otherwise) in 9.2 nl PBS containing 0.01% Tween20 using a microcapillary connected to a Nanoject II Auto-Nanoliter Injector (Drummond). The same volume of PBS-0.01% Tween20 was injected for control experiments. Experiments were performed at 25°C or 29°C depending on fly strains used.

For the feeding experiments, spores were resuspended in 100 mM sucrose solution to obtain a solution of 2.10^5^ spores/mL. Flies were exposed to 200 µL of solution that was added in Eppendorf caps, which were placed at the bottom of medium-size vials (3.5 cm diameter). A 100 mM sucrose solution was used as a control. Experiments were performed at 29°C and flies were switched back to food after one day of exposure.

### Survival Tests

Survival tests were performed using 10 to 20 flies per vial in biological triplicates per experiment. Adult flies used for survival tests were 5–7-days old from 25°C stock. For survival tests using RNAi-silencing genes, flies were kept for 7 days more at 29°C to express the RNAi prior to the experiment. Flies were counted every day. The number of independent experiments is specified in each figure legend.

### Parasite Quantification

Parasite quantification was determined using five adult flies per condition. Flies were transferred into 2-mL microtubes (Starstedt) containing five 1.4-mm ceramic beads (Dominique Dutcher) in 200 μl proteinase K solution and crushed using the Precellys 24 Tissue homogenizer (Bertin Technologies). Total genomic DNA was extracted using the NucleoSpin 96 Tissue kit (Macherey-Nagel) according to the manufacturer’s instructions. Samples of genomic DNA were diluted to 1/10 into milliQ water. Quantification was performed by qPCR using SYBR Green (Bio-Rad) as described previously (58). Only primer couples with over 90% efficiency were used. Data were normalized 1) using the *Drosophila* ubiquitous gene *RpL32* (encoding ribosomal protein 49) 2) using flies frozen on the day of injection and 3) using wild-type controls. Primers used for *RpL32* were the forward 5’-GACGCTTCAAGGGACAGTATCTG-3’ and the reverse 5’-AAACGCGGTTCTGCATGAG-3’. Primers used for *T. ratisbonensis* were the forward 5’- TCTCACAGTAGTGGCGAATG-3’ and the reverse 5’-AACACCGTATTGGAATACAG-3’.

### Antibody Production

Polyclonal rabbit antiserum raised against *T. ratisbonensis* spores was produced as described (74).

### Triacylglyceride Quantification

Triglycerides (TAGs) were quantified on samples of five adult flies per condition. Flies were transferred into 2 mL Micro tube (Starstedt) containing five 14 mm ceramic beads (Dominique Dutcher) and mixed using the Precellys 24 Tissue homogenizer (Bertin Technologies). Total TAGs were extracted using the Triglyceride Colorimetric Assay Kit (Cayman Chemicals, #10010303) according to the instructions of the manufacturer.

### Stainings

Fat body dissections were performed on flies expressing GFP-mCherry-Atg8a. Flies were cut transversally with a scalpel on a petri dish cleaned with 70% ethanol to observe the fat bodies. Fat bodies were fixed with PFA 8% and mounted on diagnostic microscope slides (Thermo Fisher Scientific) in Vectashield with DAPI (Vector Laboratories).

To observe hemocytes, wild-type larvae injected with untreated or heat killed spores (treatment at 100°C for 15 minutes) were opened 6 hours after injection in a drop of 1x PBS directly on diagnostic microscope slides. After dissection, samples were left for 30 minutes to settle the cells on the slides. Hemocytes were fixed with 8% PFA, permeabilized for 15 min with 1x PBS and 0.1% Triton X-100. Samples were blocked for 2h in 1x PBS, 0.1% Triton X-100 and 2% BSA (PTB). Hemocytes were incubated in PTB plus the primary rabbit antibody anti-*Tr* spores (1/500) and 10 μM of FITC phalloidin (Sigma-Aldrich #P5282). Samples were washed for 15 min with 1x PBS and 0.1% Triton X-100. Cells were incubated for 2h on PTB plus the secondary goat antibody anti-rabbit coupled to Cy3 (1/500) (Invitrogen #A10522). Hemocytes were washed for 15 min with 1x PBS and 0.1% Triton X-100 and mounted in Vectashield with DAPI (Vector Laboratories). All samples were observed using a LSM 780 confocal microscope (Zeiss).

### Fluorescence Quantification

Fluorescence was quantified using the ImageJ program. Channels were separated and analyzed by measuring the fluorescent signal intensity for each channel. For autophagy quantifications, a ratio of GFP/mCherry signal intensity was measured.

### Injection of GPIs

1 mM of chemically synthesized GPIs structures (GIcN-IP or Man_4_GIcN-IP) (75) or 10^4^ *T. ratisbonensis* spores were injected into *pDipt-LacZ* reporter flies.

### Latex Beads Injection

Adult *w^A5001^* flies were injected with 69 nL of latex beads solution to saturate hemocytes as described (76). Flies were also injected with 69 nL of 1x PBS as a control. We checked the efficiency of phagocytosis blockage by injecting pHrodo-labeled *Escherichia coli* (**Figure S5B**).

### Cytochalasin D Injection

Cytochalasin D (Sigma-Aldrich #PC8273) was resuspended in DMSO to achieve a 1 mg/mL stock solution and eventually diluted in 1x PBS to get a working solution at 20 µg/mL. Cytochalasins are metabolites obtained from fungi, which act as mycotoxins by blocking actin polymerization. We inhibited hemocytes activity by injecting 69 nL of cytochalasin D solution and mock-injected flies were injected with a PBS solution containing 2% DMSO.

### pHrodo Injection

Adult flies were injected with killed *E. coli* coupled to pHrodo Red (Thermo Fisher Scientific) as described (77). Hemocytes were stimulated by injecting 69 nL of pHrodo Red coupled *E. coli*. The fluorescence of pHrodo Red increases as pH decreases indicating when pHrodo is located into the mature phagosome with its acidic pH. At neutral or basic pH, pHrodo Red is non-fluorescent. The same volume of 1x PBS was injected as a control.

### H_2_O_2_ Measurements

H_2_O_2_ measurements were performed on 4x5 flies using the hydrogen peroxide assay kit (Sigma-Aldrich #MAK165) following the instructions provided by the supplier.

### Statistical Analyses

All graphs and statistical tests were performed using GraphPad Prism. The statistical test used for the survival curves was Log-rank. For parasite load experiments, Mann-Whitney, unpaired *t-*test or one-way ANOVA were used. For autophagy, H_2_O_2_ and TAGs quantifications experiments Kruskall-Wallis or one-way ANOVA test were performed. When performing parametric unpaired *t*-tests, a Gaussian distribution of data was checked using either D’Agostino-Pearson omnibus or Shapiro-Wilk normality tests. The number of stars (*) represents the P values P≥0.05 (ns), P<0.05 (*), P<0.01 (**), P<0.001 (***) and P<0.0001 (****).

## Results

### The Systemic Immune Response Toll & IMD Pathways Are Not Effective to Restrict *T. ratisbonensis* Infection

The injection of microorganisms within the body cavity usually triggers one or both NF-κB-type pathways; Toll or IMD. We have previously reported that we did not observe any consistent induction of these pathways by measuring the expression of AMP genes by RTqPCR, in keeping with the absence of peptidoglycan or ß-glucans in microsporidia (43, 78). However, we occasionally observed signals in some fat body lobules with a *pDipt-LacZ* reporter transgene but not with a *pDrosomycin-GFP* reporter (**Figure S1A**). We therefore wondered whether other constituents of the spore cell wall might be detected, albeit weakly, by the innate immune system. A class of compounds present on the surface of parasites detected by the vertebrate innate immune system are glycosylphosphatidyl-inositols (GPIs) that anchor some surface proteins to the cytoplasmic membrane. We therefore wondered whether GPI anchors might also elicit the *Drosophila* systemic immune response. To identify the type of GPIs potentially present on the surface of *T. ratisbonensis* spores, we have incubated a *T. ratisbonensis*-specific antiserum on a chip that displays some synthetic GPIs (**Figure S1B**). Whereas sera directed against the human-infecting microsporidia species yielded a weak positive signal with EtN-Man_4_GlcN-PI, we observed a strong signal with two epitopes, GlcN-PI and Man_4_GlcN-IP as well as milder signals with some other GPIs (**Figure S1C**). The injection of the two synthetic GPIs that yielded a strong signal did not however induce a consistent expression of the *pDipt-LacZ* reporter transgene as observed after an *Escherichia coli* challenge (**Figures S1A, D**).

**Figure 1:**
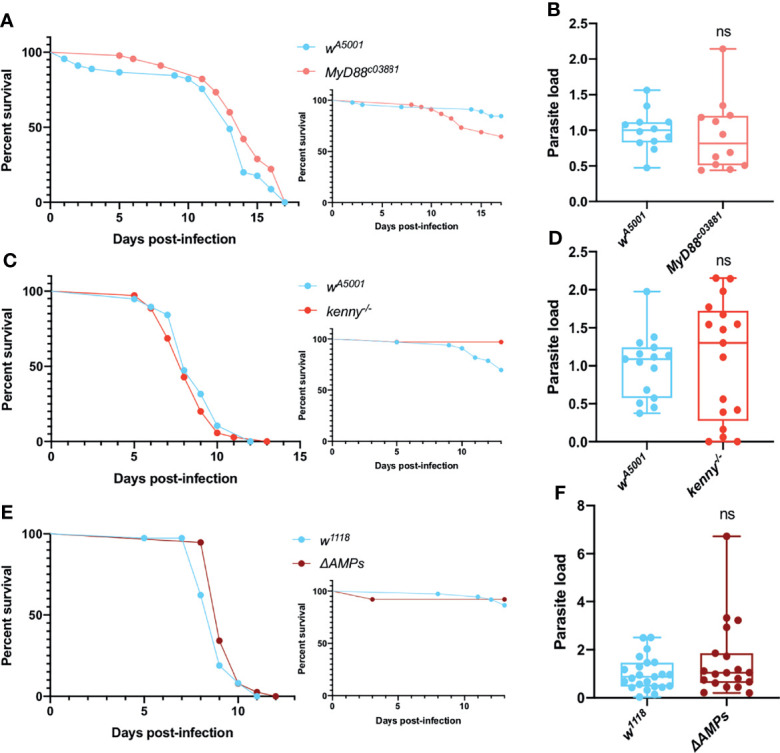
Toll and IMD pathways are not involved in parasite control. **(A, B)** Survival curve and relative parasite load measured by qPCR at 9 days post-infection of isogenized *Myd88^c03881^* mutant flies infected by *T. ratisbonensis*. **(C, D)** Survival curve and relative parasite load measured by qPCR at 6 days post-infection of *kenny* mutant flies infected by *T. ratisbonensis*. **(E, F)** Survival curve and relative parasite load measured by qPCR at 6 days post-infection of *ΔAMPs* mutant flies infected by *T. ratisbonensis*. The silencing of genes involved in the Toll & IMD pathways was not impacting either fly survival or parasite loads, which was confirmed by testing the *ΔAMPs* mutant. Experiments were performed at 25°C **(A, B)** or 29°C **(C-F)** on initially 5-7 day-old female flies. The inset graphs display survivals of control noninfected flies injected with PBS. Each survival graph is representative of at least three independent experiments. Parasite load graphs represent the pooled data of at least three independent experiments. Survival data were analyzed using the log-rank statistical test. qPCR data were analyzed using an unpaired *t-*test. ns, not significant.

We next examined the survival and parasite titers of Toll pathway mutant *MyD88^c03881^* flies following the injection of *T. ratisbonensis*. *MyD88^c03881^* infected flies were not more sensitive to the infection compared to wild-type flies (**Figure 1A**) and both wild-type and mutant flies exhibited a similar parasite load (**Figure 1B**). The other major NF-κB pathway in *Drosophila*, Immune deficiency (IMD), also did not appear to be involved in the host defense against *T. ratisbonensis* since *kenny* mutant and wild-type flies displayed similar survival curves and parasite burden (**Figures 1C, D**). Additionally, we decided to test a mutant fly line in which all major known AMP genes have been deleted (65). The survival and parasite load of these *ΔAMPs* mutant infected flies were similar to those of wild-type flies (**Figures 1E, F**).

Taken together, these data suggest that the Toll and IMD pathways do not significantly contribute to *Drosophila* host defense against *T. ratisbonensis*.

### Ambiguous Roles of Oxidative Stress and Stress Response Pathways in Host Defense Against *T. ratisbonensis*

**Figure 2:**
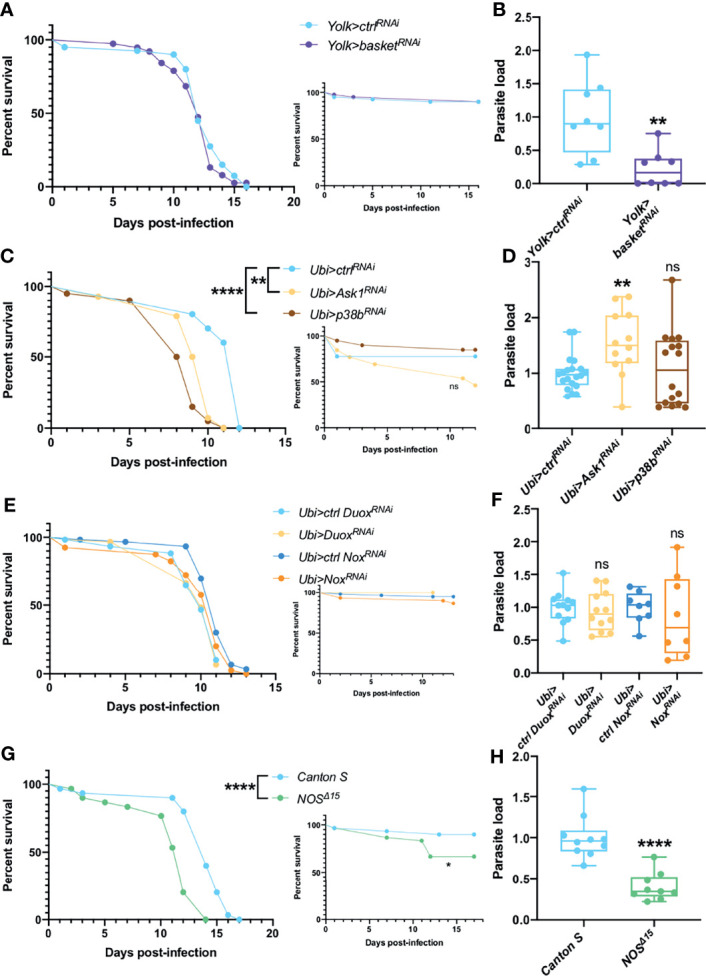
JNK, ROS, NOS & NOX are not involved in parasite control. **(A, B)** Survival test and relative parasite load measured by qPCR at 6 days post-infection of flies infected with *T. ratisbonensis* after *basket* RNAi knockdown driven in the fat body using a *Yolk-Gal4* driver. Downregulating the expression of *basket* did not affect fly survival, but decreased the parasite load. **(C, D)** Survival test and relative parasite load measured by qPCR at 6 days post-infection of flies infected with *T. ratisbonensis* after *Ask1* or *p38b* RNAi knockdown in the whole fly using a *Ubi-Gal4-Gal80^ts^* driver. Downregulating the expression of *Ask1* or *p38b* negatively affected fly survival but only the RNAi knockdown of *Ask1* increased the parasite load. **(E, F)** Survival test and relative parasite load measured by qPCR 6 days post-infection of flies infected with *T. ratisbonensis* after *Duox* or *NOX* knockdown using a *Ubi-Gal4-Gal80^ts^* driver. **(G, H)** Survival test and relative parasite load measured by qPCR 9 days post-infection of *NOS^Δ15^* mutant flies infected with *T. ratisbonensis*. Silencing of *Duox* or *NOX* did not reveal any immune defense phenotype but *NOS^Δ15^* mutant flies exhibited higher sensitivity in survival (4/4 experiments) and lower loads. Experiments were performed at 29°C **(A-F)**, except for experiments shown in G-H that were done at 25°C, on initially 5-7 day-old female flies. The inset graphs display survivals of control non-infected flies injected with PBS. Each survival graph is representative of at least two to three independent experiments, except for *Ask1* and *p38b*, as survivals were highly variable **(Figures S2A, B)**. Parasite load graphs represent the pooled data of at least two independent experiments. Survival data were analyzed using the log-rank statistical test. qPCR data were analyzed using an unpaired *t-*test. The number of stars (*) represents the P values P≥0.05 (ns), (**), and P<0.0001 (****). ns, not significant.

Physical or biological stresses such as exposure to ROS or infections are also known to activate MAPKs pathways in *Drosophila*. We first silenced by RNAi the Jun kinase (JNK) gene *basket* in the fat body. Even though the silenced flies behaved as control flies in survival assays (**Figure 2A**), they consistently exhibited a decreased parasitic burden (**Figure 2B**). In contrast, when ubiquitously knocking-down the apoptotic signal-regulating kinase 1 *Ask1* or the MAPK *p38b*, variable survival curves were observed, from increased sensitivity to improved resistance against *T. ratisbonensis* infection (*Ask1*) or increased sensitivity to no phenotype (*p38b*) (**Figure 2C** and **Figures S2A, B**). The parasite load was equal in *p38b* knockdown flies whereas it was somewhat increased after *Ask1* knockdown (**Figure 2D**).

We have also tested whether enzymes that generate ROS or Reactive Nitrogen Species such as the Nitric Oxide Synthase NOS are involved in the host defense against *T. ratisbonensis*. The ubiquitous silencing of NADPH-oxidase genes *Duox* or *Nox* in the whole fly had no significant impact on fly survival and parasite load (**Figures 2E, F**) and quantifying H_2_O_2_ levels upon infection did not present any phenotype (**Figure S2C**). However, *NOS* null mutants were more susceptible to the infection (**Figure 2G**). Unexpectedly, the parasite load was significantly decreased (**Figure 2H**). Taken together, these data suggest that the ROS response is not involved in parasite control. In contrast, there is an uncoupling between the phenotypes of survival and parasite loads when the *basket*, *Ask1-p38* or the *NOS* genes are affected.

### Xenophagy Does Not Control *T. ratisbonensis* Infection

Autophagy is involved in the elimination of damaged endogenous components such as defective mitochondria as well as the removal of exogenous material in a process called xenophagy when dealing with invading pathogens. In *Drosophila*, it has been shown that *L. monocytogenes* is inducing xenophagy through PGRP-LE recognition (46). As microsporidia are intracellular parasites and proliferate mostly in fat body cells by stealing lipids (43), autophagy might be involved in parasite control either directly by xenophagy or indirectly by regulating the access to lipid stores by lipophagy.

**Figure 3:**
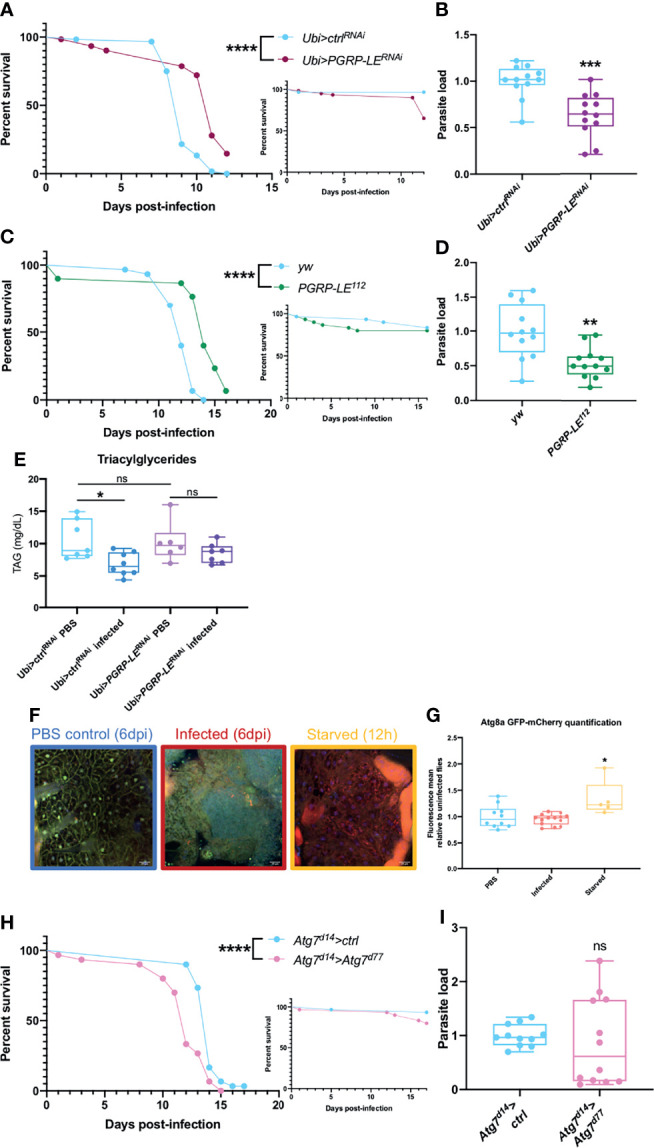
Xenophagy is not involved in parasite control. **(A, B)** Survival test and relative parasite load measured by qPCR 6 days post-infection of flies infected with *T. ratisbonensis* after *PGRP-LE* knockdown using a *Ubi-Gal4-Gal80^ts^* driver. **(C, D)** Survival test and relative parasite load measured by qPCR 9 days post-infection of *PGRP-LE^112^* mutant flies infected with *T. ratisbonensis*. Performing a knockdown of *PGRP-LE* or using a mutant line improved fly survival and reduced parasite loads. **(E)** TAGs quantification on *PGRP-LE* knockdown infected flies. **(F)** Confocal pictures of GFP-mCherry-Atg8a uninfected flies 6 days post-injection (left), infected with *T. ratisbonensis* 6 days post-infection (middle), or starved flies after 12 hours on water (right). Blue = nuclei; yellow = autophagosomes; red = autolysosomes **(G)** Quantification of the ratio of mCherry fluorescence over GFP fluorescence (flies from the experiment shown in **F**). **(H, I)** Survival and relative parasite load measured by qPCR at 7 days post-infection of *Atg7* trans heterozygous mutant flies infected by *T. ratisbonensis*. Autophagy was not induced upon *T. ratisbonensis* infection and flies lacking functional autophagy were more sensitive to the infection, which was not correlated with a higher load. Experiments were performed at 29°C except for experiments shown in **(C, D)** that were done at 25°C, on initially 5-7 days old female flies. The inset graphs display survivals of control non-infected flies injected with PBS. Each survival graph is representative of at least three independent experiments. Parasite load graphs represent the pooled data of at least three independent experiments. Autophagy flux quantification graph is a merge of two independent experiments. TAG graph represents the pooled data of two independent experiments. Survival data were analyzed using the log-rank statistical test. qPCR data were analyzed using an unpaired *t-*test. Autophagy quantification was analyzed using a Kruskall-Wallis test. TAG graph was analyzed using one-way ANOVA statistical test. The number of stars (*) represents the P values P≥0.05 (ns), P<0.05 (*), P<0.01 (**), P<0.001 (***) and P<0.0001 (****). ns, not significant.

To study the role of PGRP-LE we used two different strategies. We silenced *PGRP-LE* expression in the whole fly and we used *PGRP-LE^112^* null mutant flies. For both type of mutations, we observed an increased resistance to infection that correlated well with a decreased parasitic burden (**Figures 3A–D**). As *T. ratisbonensis* proliferates by preying onto host lipids, we also checked triacylglyceride (TAG) levels. The TAG reserves were intact in uninfected *PGRP-LE* silenced flies. Unexpectedly, whereas control flies effectively exhibited depleted TAG stores upon *T. ratisbonensis* infection, this was no longer the case when *PGRP-LE* was silenced (**Figure 3E**). One interpretation of these data is that PGRP-LE is required by the parasite to hijack host lipid reserves.

As PGRP-LE is connected to autophagy and as autophagy has been reported to be an intracellular host defense against microsporidia in *C. elegans* (28, 79), we next used a fly reporter *GFP-mCherry-Atg8a* line (61) to assess whether any autophagic vacuoles form upon invasion by *T. ratisbonensis*. We did not observe any autophagy induction in microsporidia infected flies compared to uninfected control (**Figures 3F, G**). To further exclude a role for autophagy during infection, we also tested an *Atg7^d14^/Atg7^d77^* transheterozygous mutant line and performed survival and parasite quantification. Even though these mutant flies were more sensitive to starvation (**Figure S3A**), as expected, and to *T. ratisbonensis* infection (**Figure 3H**), *Atg7* mutant flies displayed an unaltered parasite burden (**Figure 3I**). To exclude a potential developmental effect in the *Atg7^d14^/Atg7^d77^* (62), we silenced *Atg7* expression solely in the adult fat body. The survival and parasite load of silenced flies was not impaired upon infection compared to control flies (**Figures S3C, D**), yet, these silenced flies were more sensitive to starvation (**Figure S3B**).

These results allow us to conclude that *T. ratisbonensis* proliferation is not controlled by xenophagy in *Drosophila*. Surprisingly, the parasite needs PGRP-LE to proliferate within the fat body cells, potentially by allowing an efficient depletion of lipidic stores.

### Antiviral Pathways Are Not Able to Control Microsporidia

Like viruses, microsporidia are obligate intracellular pathogens. We therefore checked for a potential involvement of antiviral pathways. One of the major antiviral defense in *Drosophila* is RNAi (47) mediated by Dicer-2. A *Dicer-2* null mutant line appeared to be more sensitive to a *T. ratisbonensis* challenge than a control rescued line (**Figure S4A**); yet, this may reflect a sensitivity to wounding as PBS-injected flies displayed a similar behavior (**Figure S4A**, inset). Indeed, the parasite load was equal in the *Dicer-2* mutant flies compared to rescued flies (**Figure S4B**). We conclude that RNA interference is not involved in parasite control.

The cGAS-STING pathway is also involved in the control of intracellular pathogens such as virus or bacteria, through the recognition of nucleic acids. Recently, the antiviral function of the *Drosophila* ortholog of STING against picorna-like viruses in *Drosophila* has been shown (48–50). *dSTING* null mutant flies were not more sensitive to infection compared to wild-type flies and their parasite burden was unaltered (**Figures S4C, D**). Altogether, we failed to obtain any evidence for an involvement of intracellular antiviral pathways in the host defense against *T. ratisbonensis*.

### Pro-Phenoloxidases (PPOs) Are Required for Parasite Proliferation

**Figure 4:**
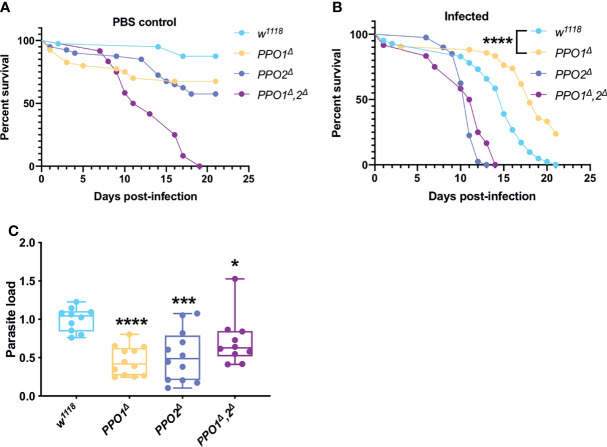
PPOs are required for parasite growth. **(A)** Survival test of *PPO1^Δ^*, *PPO2^Δ^*, and *PPO1^Δ^*-*PPO2^Δ^* mutant flies injected with PBS. **(B ,C)** Survival tests and relative parasite loads measured qPCR of *PPO1^Δ^*, *PPO2^Δ^*, and *PPO1^Δ^*-*PPO2^Δ^* mutant flies infected by *T. ratisbonensis*. *PPO* mutant flies were slowly succumbing from PBS injection; however, *PPO1^Δ^* mutant flies were more resistant to the infection and this was correlated with a lower parasitic burden, a phenotype found for all *PPO* mutants. All experiments were performed at 25°C on initially 5-7 day-old female flies. Parasite loads were performed at 9 days post infection. Each survival graph is representative of at least three independent experiments. Parasite load graphs represent the pooled data from three independent experiments. All experiments were also performed using another wild-type control and yielded similar results. Survival data were analyzed using the log-rank statistical test. qPCR data were analyzed using an unpaired *t-*test. The number of stars (*) represents the P values P≥0.05 (ns), P<0.05 (*), P<0.001 (***) and P<0.0001 (****).

Melanization is one of the major host immune response in insects (80). This pathway is dependent on key enzymes: the phenoloxidases (POs), catalyzing the oxidation of phenols to quinones and ultimately polymerizing into melanin at the wounding site. POs are first synthesized as inactive zymogens called proPOs (PPOs) prior to being cleaved to generate active POs. The survival phenotypes of *PPO1* and *PPO2* mutant flies were opposite, with the former being more resistant and the latter more sensitive to *T. ratisbonensis* infection (**Figure 4B**). We note that both mutants exhibit a late, mild sensitivity to injury (**Figure 4A**). In contrast, the PPO1-PPO2 double mutant was highly sensitive to “clean” wounds in this series of experiments precluding an interpretation of its survival curve when infected (**Figures 4A, B**). Unexpectedly, the microsporidial burden was lower than in wild-type for all *PPO* mutants (**Figure 4C**).

Thus, even though *PPO2* appeared to be required in host resilience against *T. ratisbonensis*, the lack of enhanced parasite load is not consistent with such a conclusion. In contrast, *PPO1* is required for an efficient *T. ratisbonensis* infection, a puzzling inference.

### Phagocytosis Is an Essential Defense to Control Microsporidia

**Figure 5:**
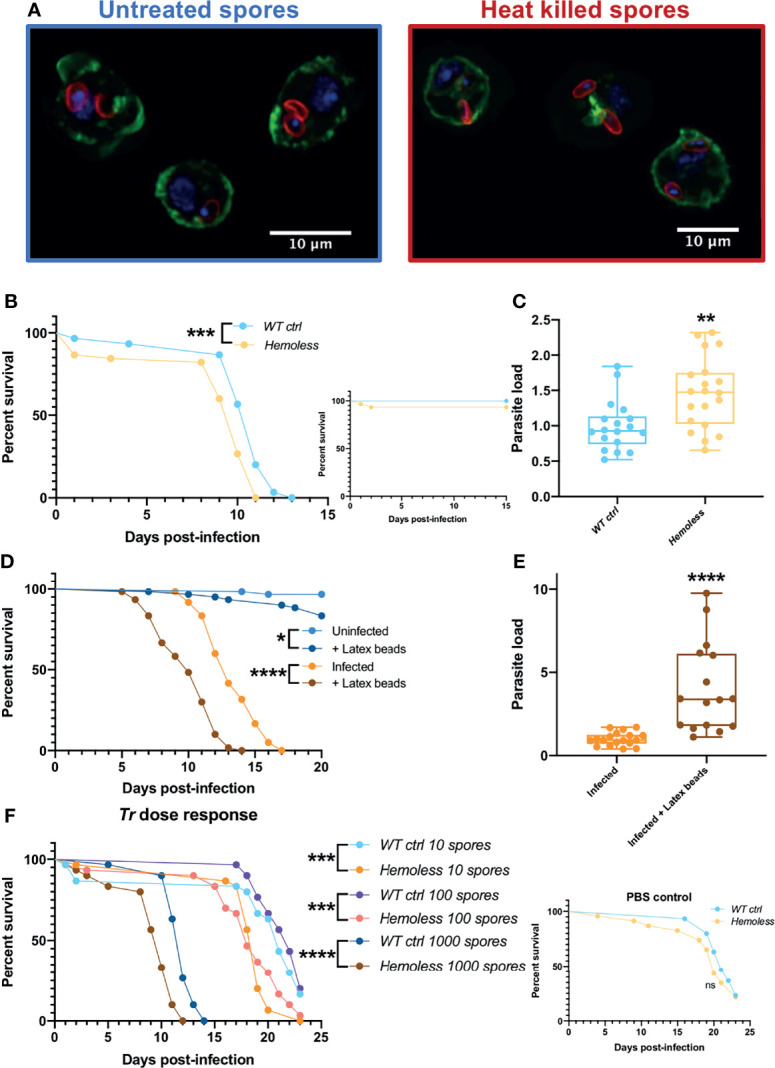
Phagocytosis provides a degree of protection against *T. ratisbonensis* infections. **(A)** Confocal pictures of hemocytes extracted from infected larvae with *T. ratisbonensis* untreated spores (left) or heat killed (right). Blue = nuclei; green = actin; red = spores **(B, C)**, Survival test and relative parasite load measured by qPCR of *T. ratisbonensis*-infected flies in which apoptosis of hemocytes was induced by crossing an *Hml-Gal4-UAS-GFP* driver with a *UAS-rpr; UAS-hid* line (*hemoless* flies). **(D, E)** Survival curves and relative parasite load measured by qPCR of flies injected with latex beads to saturate the phagocytic apparatus. **(F)** Survival curves of *hemoless* flies injected with low doses of spores (10 to 1000 spores). Blocking phagocytosis, genetically or mechanically, always impaired fly survival and correlated with a higher parasitic burden, indicating a role in resistance to *T. ratisbonensis* infection. All experiments were performed at 29°C on initially 5-7 day-old female flies. The inset graphs display survivals of control non-infected flies injected with PBS. Parasite loads were performed 6 days post-infection. Each survival graph is representative of at least three independent experiments. Parasite load graphs represent the pooled data of at least three independent experiments. Survival data were analyzed using the log-rank statistical test. qPCR data were analyzed using an unpaired *t-*test. The number of stars (*) represents the P values P≥0.05 (ns), P<0.05 (*), P<0.01 (**), P<0.001 (***) and P<0.0001 (****).

In adults, the cellular immune response mainly eliminates invading pathogens through phagocytosis. We first checked that plasmatocytes are able to ingest lived and killed injected *T. ratisbonensis* spores using confocal microscopy on hemocytes retrieved from injected larvae (**Figure 5A**). We next used several functional approaches to inactivate phagocytosis in adults. First, we depleted hemocytes in adults by inducing their apoptosis throughout development (**Figure S5A**). These “hemoless” flies were somewhat more sensitive to infection and displayed an increased microsporidial titer (**Figures 5B, C**; **Figure S5B**). Similar results were obtained upon the saturation of the phagocytic apparatus by the prior injection of nondegradable latex beads that are phagocytosed by plasmatocytes (**Figures 5D, E**). The injection of cytochalasin also blocks phagocytosis and in keeping with the previous results led to an enhanced sensitivity to *T. ratisbonensis* infection (**Figure S5C**).

Wild-type flies do succumb within 10-15 days to the injection of 2000 spores as documented in all experiments shown so far. They also succumb at the same rate to the injection of 1000 spores (**Figure 5F**). However, when they were injected with lower doses such as 10 or 100 spores on average, they succumbed at the same rate as PBS-injected controls (**Figure 5F** and inset). In contrast, “hemoless” flies injected with low doses were killed at a significantly faster pace (**Figure 5F**). These data indicate that the cellular immune response can control low intensity infections but becomes overwhelmed when exposed to higher inocula.

### The Uptake of Microsporidia Does Not Require Most Known Phagocytic Receptors

Several potential phagocytosis receptors that mediate the uptake of microbes or apoptotic bodies have been identified in *Drosophila* (68, 81–85).

We first tested mutant lines for *eater*, *NimA*, and *Draper.* The survival and parasitic loads of *eater* and *NimA* mutants were comparable to wild-type controls infected with *T. ratisbonensis* spores (**Figures S6A–D**). Unexpectedly, the survival of *Drpr^HP37013^* mutant infected flies was improved compared to wild-type controls (**Figure S6E**) and parasite load was decreased (**Figure S6F**), which suggests that Draper promotes *T. ratisbonensis* infection.

We relied on RNA interference induced only at the adult stage to test other potential phagocytosis receptors (or opsonins). Several lines did not display an altered survival to a *T. ratisbonensis* challenge, those targeting *NimB1*, *NimB2*, *croquemort* (*crq*), and one *NimB4* RNAi line (**Figure S6G**). Three lines appeared to be more resistant to *T. ratisbonensis* infection, *i.e.*, those affecting *peste* (*pes*), *NimB5*, and *NimB4* (a second RNAi line). No conclusion can be drawn as regards *NimC1* as mock-infected controls died as rapidly as *T. ratisbonensis*-infected RNAi flies. With respect to the microsporidial burden, a trend toward a reduced *T. ratisbonensis* titer was observed for *NimB5*, *NimB4*, and *crq* (**Figure S6H**), although the survival of the latter two RNAi lines was not affected. For most other RNAi lines, the measured load was variable from experiment to experiment, as exemplified by the observed bimodal distribution for *NimB1, NimB2, NimB4* (the sensitive line), *NimC1*, and *pes* (**Figure S6H**). Thus, the results are difficult to interpret reliably, even though the titers for the *pes* RNAi line was statistically significant but not correlating to their survival phenotypes (no enhanced sensitivity or protection).

Thioester-containing proteins (TEPs) belong to the superfamily of complement-like factors and have been shown in some instances to act as opsonins in insects (86, 87). We have tested *Drosophila* null mutant lines affecting either individual *Tep* genes or removing all of them, except *Tep6*, which has been shown to function in intestinal epithelium barrier function (88, 89). The individual *Tep2*, *Tep3*, and *Tep4* lines were isogenized in the *w^A5001^* genetic background while the *Tep1* and compound deletion mutant *Tep-q^Δ^* were isogenized in the Drosdel *w^1118^* background. All the mutant lines displayed a survival and parasitic burden that were similar to those of their respective controls, except for *Tep3* that was slightly more resistant to *T. ratisbonensis* infection (**Figure S7**).

In conclusion, we have not identified so far the receptors or putative opsonins that may be required for the uptake of microsporidia by plasmatocytes.

### Hemocyte Signaling Does Not Impact Parasite Proliferation But May Improve Fly Resilience to *T. ratisbonensis* Infection

We cannot formally exclude that the approaches that we have used to block phagocytosis may also impede other functions of hemocytes such as cytokine signaling.

**Figure 6:**
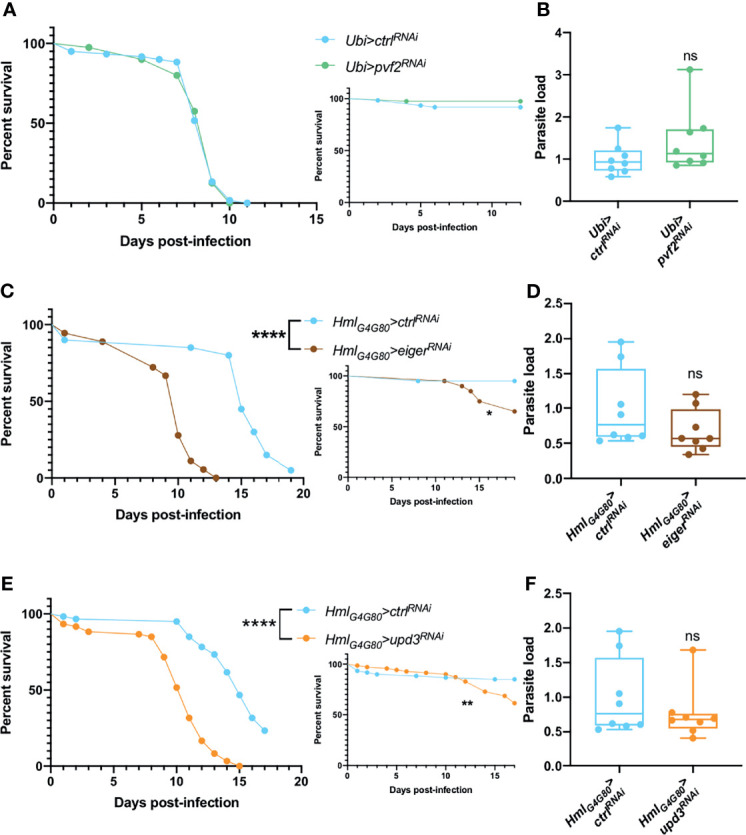
Hemocyte signaling might be involved in fly resilience upon *T. ratisbonensis* infection. **(A, B)** Survival test and relative parasite load measured by qPCR of flies infected with *T. ratisbonensis* after *pvf2* knockdown using a *Ubi-Gal4-Gal80^ts^* driver. **(C, D)** Survival test and relative parasite load measured by qPCR of flies infected with *T. ratisbonensis* after *eiger* knockdown using a *Hml-Gal4-Gal80^ts^* driver. **(E, F)** Survival test and relative parasite load measured by qPCR of flies infected with *T. ratisbonensis* after *upd3* knockdown using a *Hml-Gal4-Gal80^ts^* driver. Survivals and loads performed after the knockdown of *pvf2* did not shown any phenotype; however, downregulating *eiger* or *upd3* expression strongly impaired the survival of infected flies compared to wild-type and non-infected controls, without impacting the parasite titer. All experiments were performed at 29°C on initially 5-7 day-old female flies. The inset graphs display survivals of control non-infected flies injected with PBS. Parasite loads were performed at 6 days post infection. Each survival graph is representative of two independent experiments. Parasite load graphs represent the pooled data of two independent experiments. Survival data were analyzed using the log-rank statistical test. qPCR data were analyzed using an unpaired *t-*test. The number of stars (*) represents the P values P≥0.05 (ns), P<0.05 (*), P<0.01 (**), and P<0.0001 (****).

A recent study has documented that *Drosophila* pupal macrophages cross the blood-brain barrier upon receiving a PDGF-like factor, Pvf2, signal from glial cells that is induced by infection (90). Such signals have also been described for the developmental migration of hemocytes and their invasion of the embryonic epithelium (91, 92). We therefore ubiquitously silenced *pvf2* in adult flies and tested their survival and microsporidial burden upon *T. ratisbonensis* infection and did not observe any difference when compared to control flies (**Figures 6A, B**).

We next tested the roles of Eiger, a ligand that activates the JNK pathway (93, 94) and UPD3, a cytokine involved in JAK-STAT activation and highly expressed in hemocytes upon septic injury (95). Upon inhibiting *eiger* or *upd3* expression specifically in hemocytes by RNAi, we observed a strongly enhanced susceptibility to *T. ratisbonensis* infection (**Figures 6C, E**) that however did not correlate with an increased parasitic load (**Figures 6D, F**).

Thus, the silencing of either *eiger* or *upd3* in hemocytes yielded the strongest phenotype observed so far in survival experiments, which underscores their importance in the host defense against *T. ratisbonensis*. The observation that the microsporidial burden is not affected in these mutants argues that these genes are required in resilience and not resistance to *T. ratisbonensis* infection.

## Discussion

We have here systematically tested in a *T. ratisbonensis* systemic infection model the known *Drosophila* antimicrobial host defenses using a genetic approach. Contrary to our expectations, we found that only phagocytosis is able to confer a degree of protection against this microsporidial infection (**Table 2**). Several other host defenses were either not relevant or unexpectedly promoted the infection by the parasite (**Table 2**). Our work therefore provides a novel perspective on insect host defenses against one obligate intracellular parasite that differs from the picture gained through descriptive transcriptomic or proteomic analyses (51, 52).

**Table 2:** Summary of the phenotypes obtained in this study.

Mutant	Survival	Load	Comments
Systemic immune response			
Myd88	=	=	
kenny	=	=	
Cellular stress responses			
basket	=	↘	
Ask1	↗ or ↘	↗	
p38b	↘ or =	=	
Duox	=	=	
NOX	=	=	
NOS	↘	↘	Signaling in resilience
Autophagy & PGRP-LE			
PGRP-LE	↗	↘	
Autophagy	=	=	
Antiviral defenses			
Dicer-2	=	=	
dSTING	=	=	
Melanization			
PPO1	↗	↘	
PPO2	↘	↘	
Cellular responses			
Phagocytosis	↘	↗	
Eater	=	=	
NimA	=	=	
Draper	↗	↘	
NimB1	=	=	
NimB2	=	variable	
NimB4	↗	↘ or =	
NimB5	↗	↘	
NimC1	?	↗	
crq	=	↘	
Peste	↗	variable	
Pvf2	=	=	
Eiger	⇩	=	
Upd3	⇩	=	

Promotes infection.

Resistance.

Resilience. ↗: increased. ↘: decreased. =: unchanged. ?: unconclusive. ⇩: highly decreased.

Several host defenses do not appear to be required or to be efficient in the *Drosophila* host defense against *T. ratisbonensis* infection. This is the case for the systemic humoral immune response mediated by the Toll and IMD pathways that jointly regulate the expression of AMP genes (96). These pathways do not appear to be consistently induced and did not present any altered phenotype to a *T. ratisbonensis* challenge. Although ROS have been proposed to be important defenses against microsporidial infections, *e.g.*, infections of the intestinal epithelium of honeybees by *Nosema ceranae* (12), we did not find that the two major enzymes known to secrete ROS extracellularly, NOX and Duox, appeared to play a role against injected *T. ratisbonensis* spores. In this respect, it is important to note that by using an injection model, we bypass local epithelial barrier defenses that may very well be highly relevant in other infection models. A more definitive answer would be provided by developing a consistent larval stage infection model. The insect complement system plays a primary role against extracellular eukaryotic parasites in insects, for instance against *Plasmodium* infections in mosquitoes (97–99). Even though injected *T. ratisbonensis* spores are initially found in the hemolymph, TEP proteins do not appear to confer any protection, possibly because of the original infection mode used by microsporidia in which a polar tube is everted within seconds near or inside target cells, a time scale that may be too rapid for an effective response. In addition, the injected sporoplasm would be shielded by the polar tube from the action of such factors. Our results do not support the possibility that xenophagy contributes to *Drosophila* host defense against *T. ratisbonensis*, in as much as several other mutant lines affecting other autophagy genes failed to yield reproducible phenotypes consistent with this possibility (**Figures S8A–D**). Finally, known antiviral defenses acting intracellularly also do not appear to be involved in the protection against this parasite.

Several genes involved in various aspects of host defense displayed a mutant phenotype consistent with a proactive role in infection and not in host defense. The mutants displayed an enhanced survival rate coupled to a decreased microsporidial load. In none of the cases is it fully clear whether these gene products are actively repurposed by the parasite, for instance through secreted virulence factors, or play a passive role in a process hijacked by the parasite. While it had originally been proposed to act extracellularly, PGRP-LE appears mostly to function as an intracellular sensor of diaminopimelic type of peptidoglycan found in the cell wall of bacilli and Gram-negative bacteria that triggers IMD pathway activation (100–102). It was therefore unexpected to find a phenotype for *PGRP-LE* since peptidoglycan is not synthesized by microsporidia. A role for the IMD pathway has been ruled out (see above) and we did not find any evidence for a requirement for autophagy, a second function of PGRP-LE against intracellular DAP-type peptidoglycan containing *L. monocytogenes* (46). Our data suggest a potential role for PGRP-LE in lipid metabolism, a critical resource for parasite growth. We note that PGRP-LE did not display any TAG store alteration in mock-infected flies, thereby excluding a basal role for PGRP-LE in the regulation of lipidic reserves. We cannot however formally exclude that the lower depletion of host lipidic stores results from the lower parasitic burden in *PGRP-LE* mutants due to independent causes. One open possibility would be that microsporidia systemic infection alter the *Drosophila* microbiota and that such changes are detected through PGRP-LE, to the parasite’s advantage by an as yet unidentified process.

Several studies have reported that melanization might play a role during infection as *PPO* genes appeared to be induced by microsporidial infections (33, 103). However, the fact that a gene is induced during microsporidia invasion does not necessarily mean that it plays a role in host defense. One should keep in mind that the induction of a given gene may not necessarily reflect the induction of a host defense but a manipulation by the parasite. This might be the case for PPO1 which appears to promote *T. ratisbonensis* infection. PPO1 is produced by crystal cells in larvae and released in the hemolymph upon the rupture of the cytoplasmic membranes after a septic injury (104). How PPO1 promotes *T. ratisbonensis* infection is unclear at present. A first step would be to determine whether its function is required within hemocytes or once PPO1 is secreted in the hemolymph. It does not appear to compete with PPO2, which displays an opposite survival phenotype, because the double mutant also harbors a decreased titer of the parasite.

Draper has first been shown to be involved in the phagocytosis of apoptotic bodies during development (105). One hypothesis is that Draper might be involved in scavenging cell debris resulting from lyzed cells and therefore providing building materials for the parasite. It will be interesting to test whether its signaling function within the Src42A-Shark-Rac1 axis is required as well as determining whether receptors such as Six-microns-under that are involved in efferocytosis, the disposal of apoptotic bodies (106), are also displaying a phenotype similar to that of Draper. In this respect, NimB4 has recently been reported to also participate in efferocytosis (107) and its *Draper-*like phenotype in our study reinforces this interpretation (**Table 2**). *crq* has initially been shown to function in efferocytosis; *crq* mutants displayed a strongly decreased *T. ratisbonensis* load, yet their survival was unaltered. The scavenger receptor Peste might play a related role but its phenotype remains uncertain despite its enhanced resistance because of a variability in its *T. ratisbonensis* burden. NimB5 also promotes the proliferation of the parasite as it displays a mutant phenotype of enhanced resistance to the microsporidial infection that correlates well with a decreased titer of *T. ratisbonensis*. Although it belongs to the Nimrod superfamily, it is a secreted protein, like NimB4, that has been described to function as an adipokine in starving larvae. It then inhibits the peripheral proliferation of hemocytes as well as their adhesion. How it functions in adults remains to be determined. It is unlikely to regulate the proliferation of hemocytes in adults, which has so far not been convincingly demonstrated (108). It might be released by fat body cells that undergo a starvation-like experience as the parasite depletes its metabolic stores. Its absence in the mutant might be construed to lead to an increased adherence of hemocytes to tissues, a hypothesis difficult to support since most hemocytes are sessile in the adult. In any case, an increased adherence is expected to lower the phagocytic function of plasmatocytes. As described below, interfering with phagocytosis yielded a phenotype opposite to that of *NimB5*. It will therefore be important to determine the function of NimB5 in the adult, especially during *T. ratisbonensis* infections.

The one process providing a degree of protection against *T. ratisbonensis* infection is phagocytosis, which only delays the fatal issue when challenged with some one thousand spores. It however appears to be able to control lower doses. One interesting idea is that it may control parasites that have crossed the intestinal barrier, much like it does for ingested *Serratia marcescens* (76) or *Pseudomonas aeruginosa* (109) bacteria. This might explain why intestinal infections are not successful in adults (**Figures S9A, B**). Our experiments did not support this possibility (**Figure S9C**). Our analysis of potential phagocytosis receptors such as Eater, NimA or Peste was unsuccessful; however, an increased microsporidial burden in *NimC1* mutants might point out to redundant function with Eater (81). Although the different methods we have used to probe cellular defenses all affect the phagocytosis function of plasmatocytes, they might at the same time affect other functions of hemocytes such as the secretion of cytokines (110–112). In this respect, mutants affecting *upd3* or *eiger* exhibited a greatly enhanced susceptibility to *T. ratisbonensis* in survival experiments, even though the microsporidial burden was not changed. This phenotype is consistent with a resilience function of these cytokine genes. Further studies will allow determining in which tissues the corresponding cytokine receptors are required and thus provide some insight into the homeostatic process that allow the fly to better survive to this intracellular parasite.

The phenotypes of *NOS* and *PPO2* may also suggest a role in resilience, except that the mutants undergo a significantly reduced parasite burden. NOS might lead indirectly to the formation of reactive nitrogen species that are thought to be noxious to pathogens. This possibility is however not compatible with the decreased *T. ratisbonensis* titer measured in these mutants. Thus, it is likely that NO generated by NOS may diffuse in the organism and fulfill a signaling function as described in mammalian immune systems (113). The function of PPO2 remains enigmatic at this stage. In larvae, it forms the crystal found in crystal cells (67). The ablation of *PPO2*-expressing hemocytes might reveal whether these cells are required for this resistance-independent defense function of PPO2. The classical view of PPO-mediated melanization killing injected parasites is not relevant in the case of *T. ratisbonensis* infection. Indeed, we have failed to observe any melanin deposition on the surface of spores.

In conclusion, this study provides a largely unexpected view of classical antimicrobial host defenses, which are irrelevant or possibly diverted by the parasite for its own purpose, at least in this infection model in which *T. ratisbonensis* spores are injected directly within the hemocoel. In the long term, it would be interesting to reproduce this study in a larval infection model, which at present is not available due to the limited control of the spore load upon ingestion and the large quantity of parasites that would be required. It is an open possibility that other unknown defense mechanisms may be also at work and might be revealed upon using -omics studies. Nevertheless, the parasite appears to win the competition with the host as we are not aware of the existence of *Drosophila* lines refractory to microsporidia infections. The finding that hemocytes are required to protect the flies from such infections, at least to a degree, establish that injected spores do elicit an immune response that is not systemic.

## Data Availability Statement

The datasets presented in this study can be found in online repositories. The names of the repository/repositories and accession number(s) can be found below: https://figshare.com/, https://figshare.com/s/566ee89ed278eb6b34f3.

## Author Contributions

GC performed most of the experimental work described in this study. AF and SN initiated this study and performed early experiments. GC and DF designed the experiments, analyzed the data and wrote the manuscript. All authors contributed to the article and approved the submitted version.

## Conflict of Interest

The authors declare that the research was conducted in the absence of any commercial or financial relationships that could be construed as a potential conflict of interest.

## Publisher’s Note

All claims expressed in this article are solely those of the authors and do not necessarily represent those of their affiliated organizations, or those of the publisher, the editors and the reviewers. Any product that may be evaluated in this article, or claim that may be made by its manufacturer, is not guaranteed or endorsed by the publisher.
