# Supplementary figures and images for: Phagocytosis Is the Sole Arm of Drosophila melanogaster Known Host Defenses That Provides Some Protection Against Microsporidia Infection
Source: Front Immunol. 2022 Apr 13;13:858360. doi: 10.3389/fimmu.2022.858360 (PMC9043853; doi:10.3389/fimmu.2022.858360)

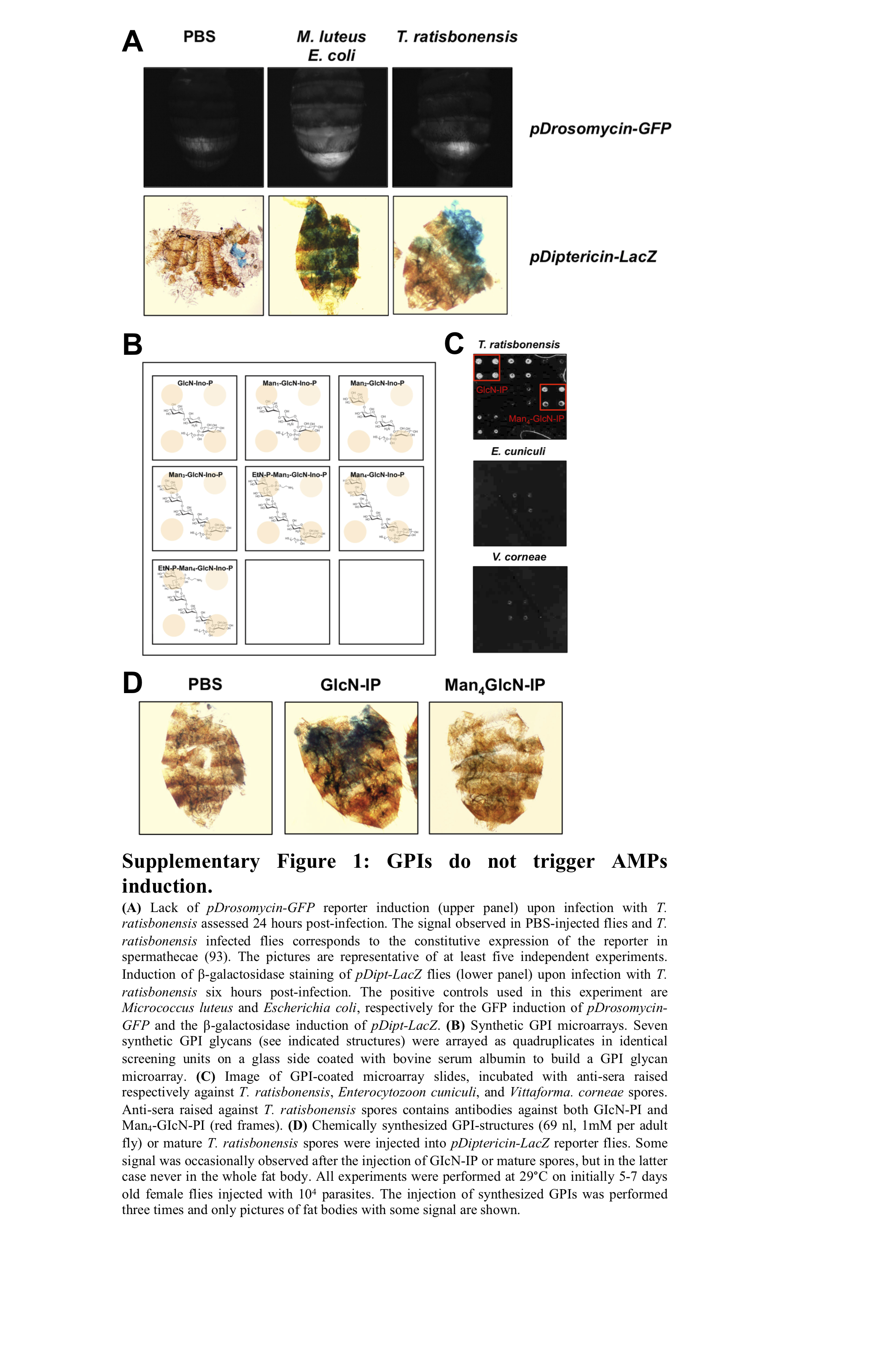

Supplement: Supplementary file 1 [file Image_1.tiff]

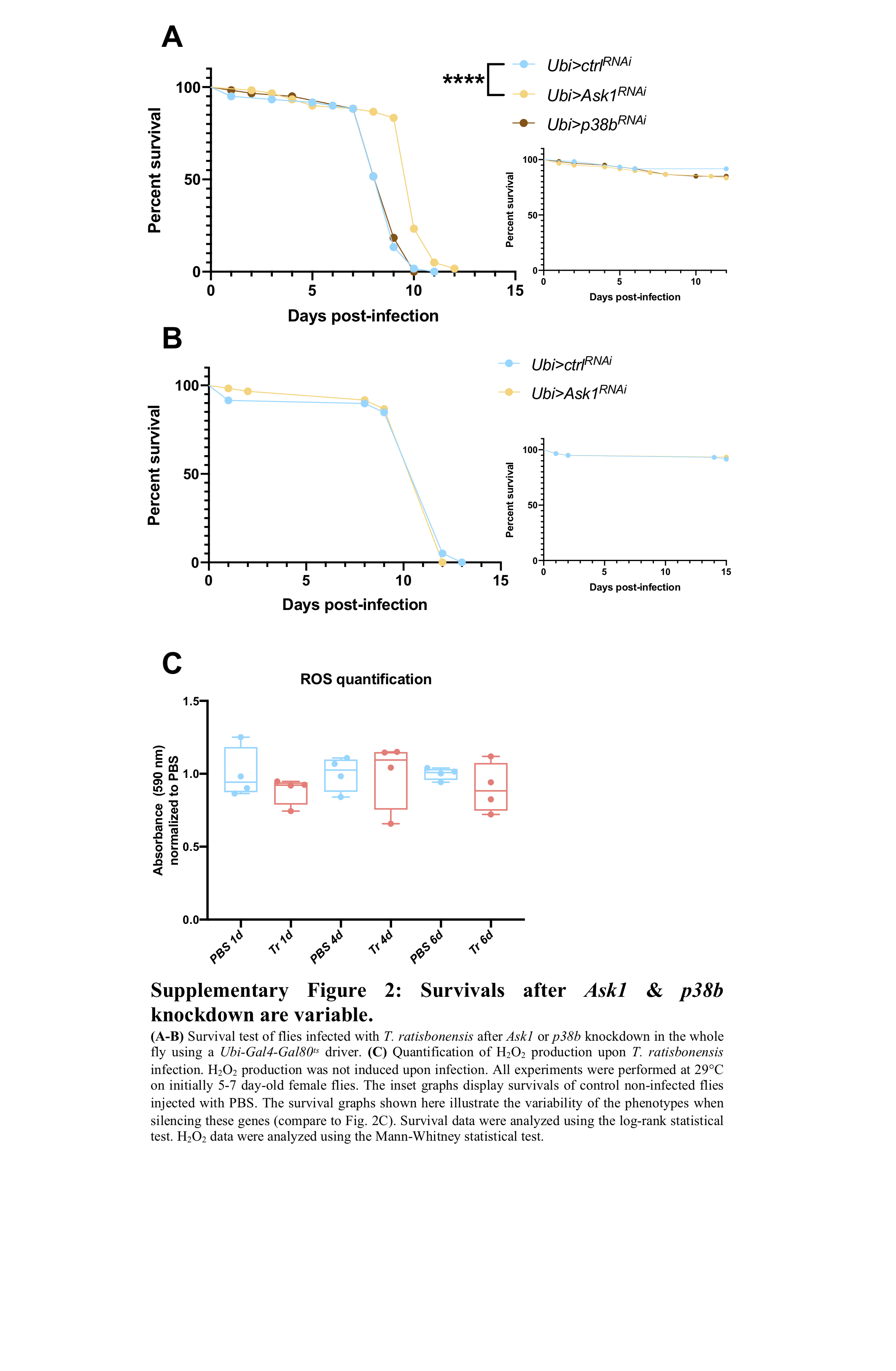

Supplement: Supplementary file 2 [file Image_2.tiff]

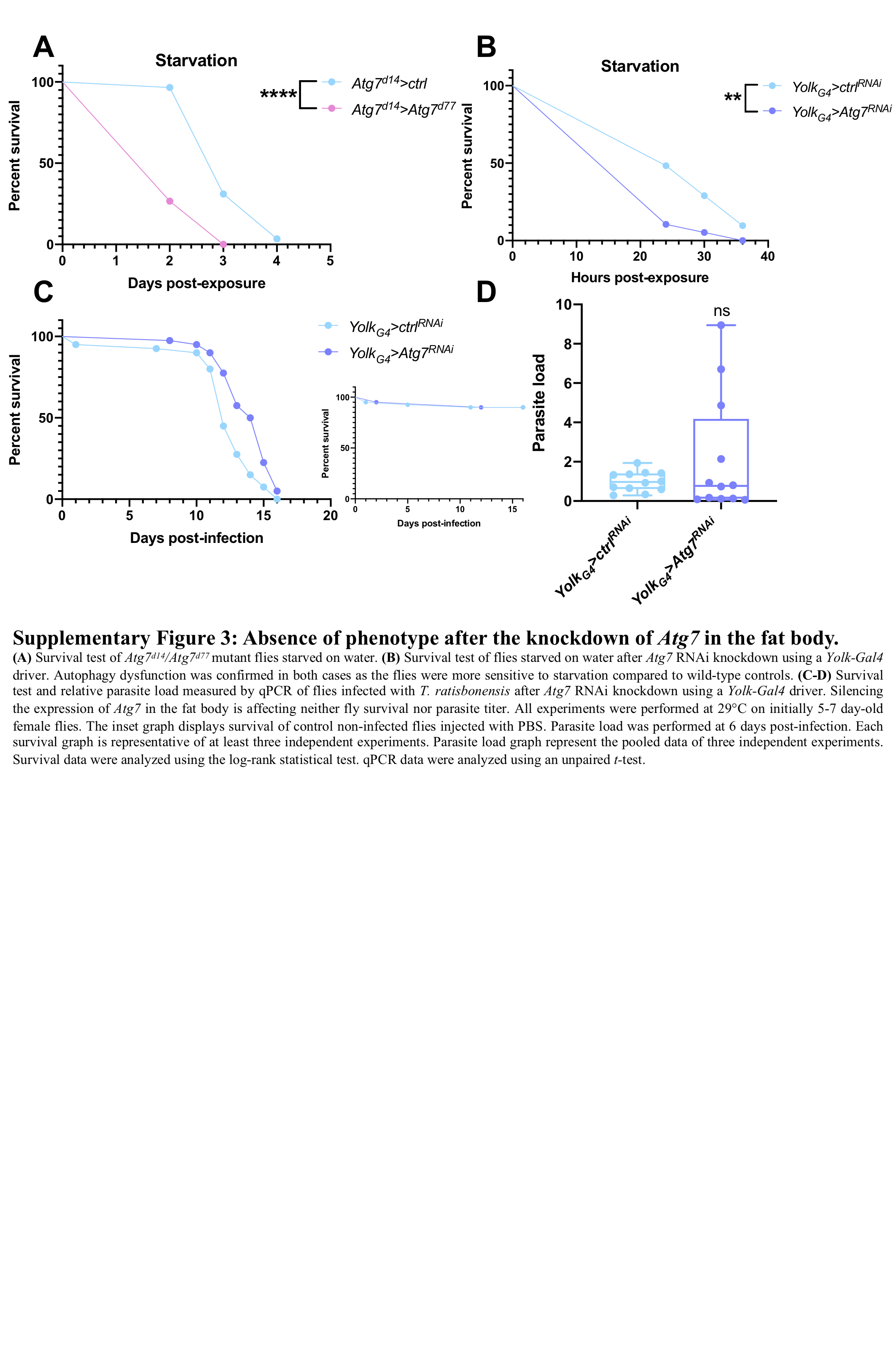

Supplement: Supplementary file 3 [file Image_3.tiff]

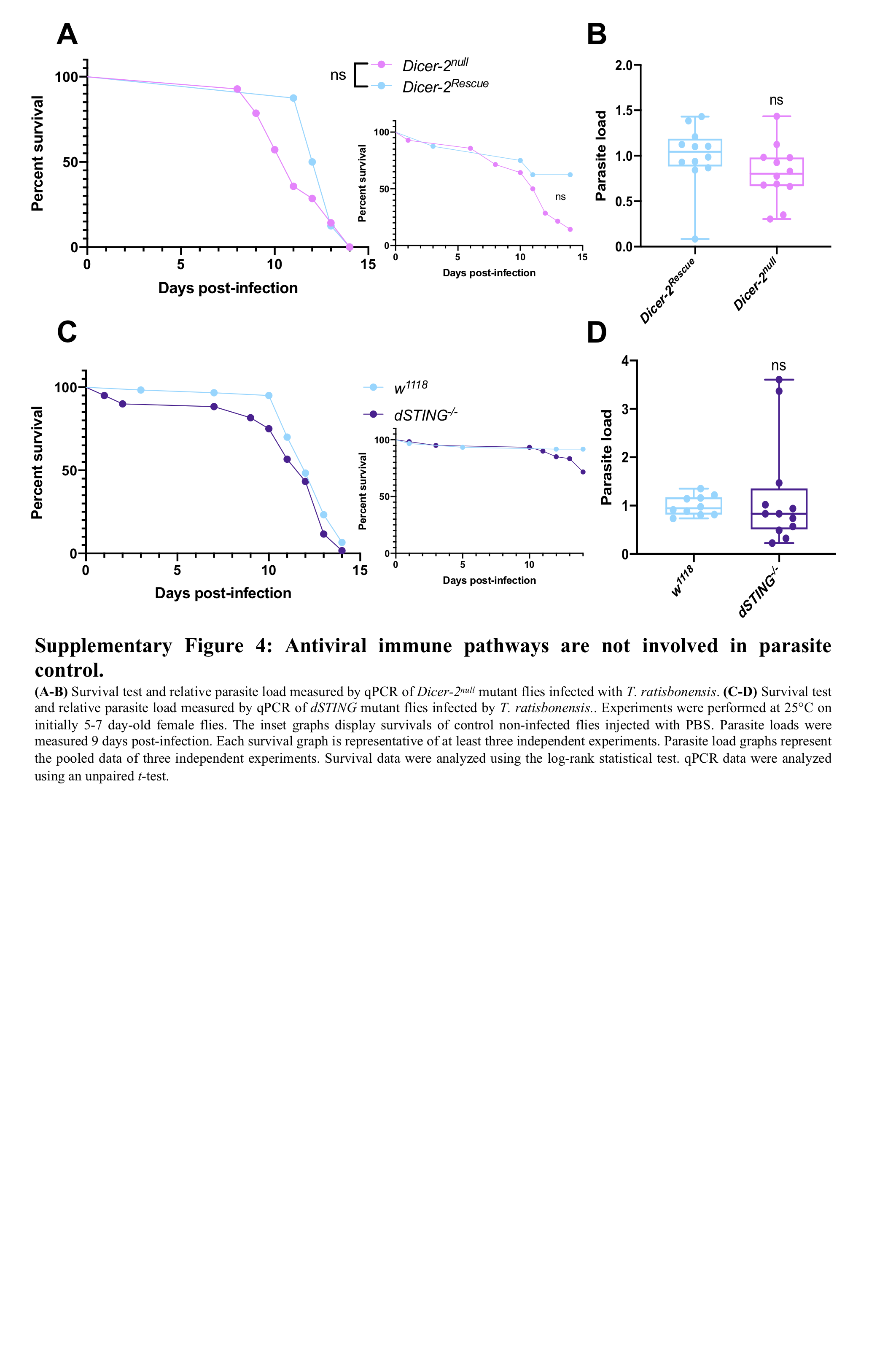

Supplement: Supplementary file 4 [file Image_4.tiff]

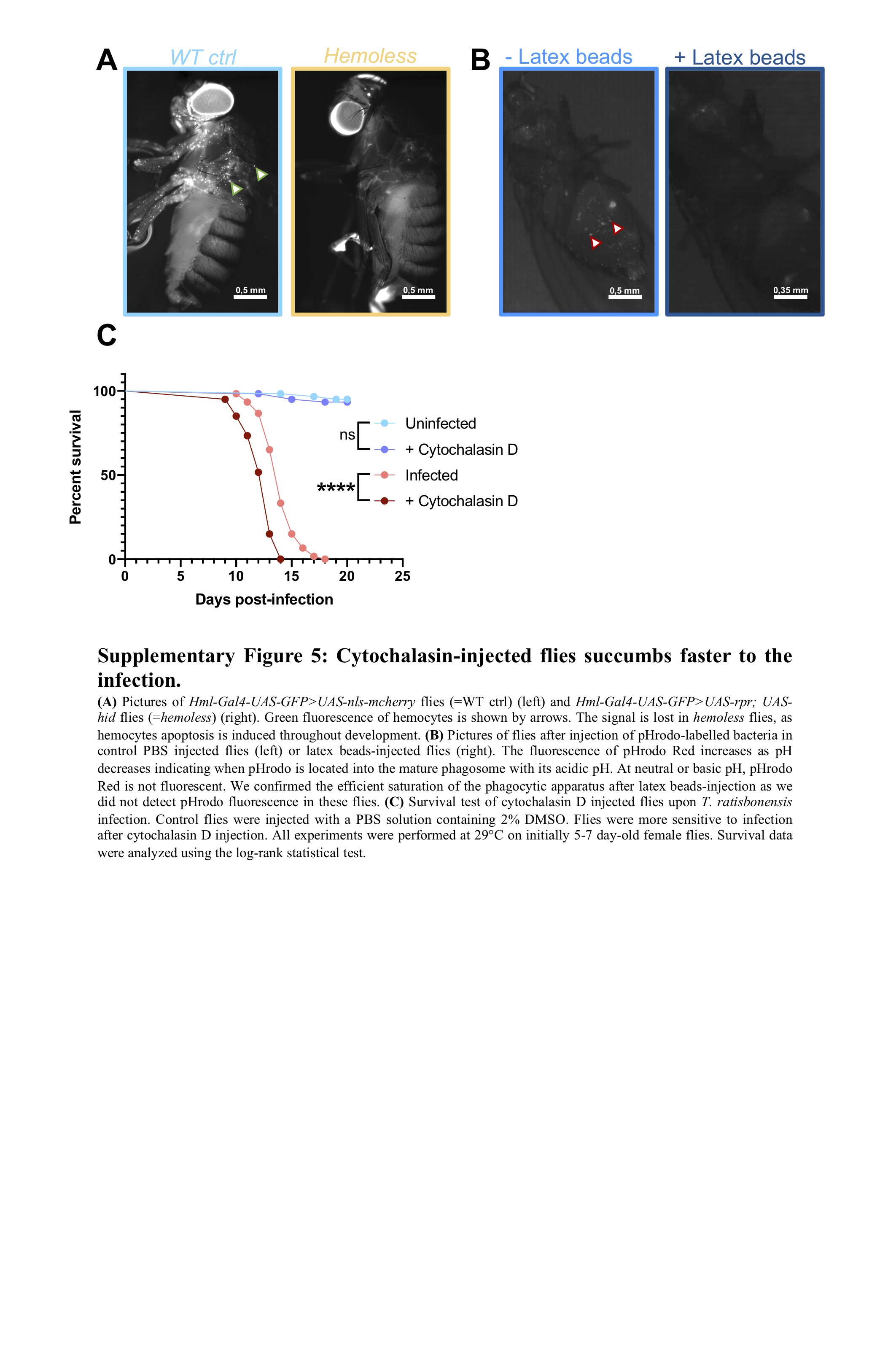

Supplement: Supplementary file 5 [file Image_5.tiff]

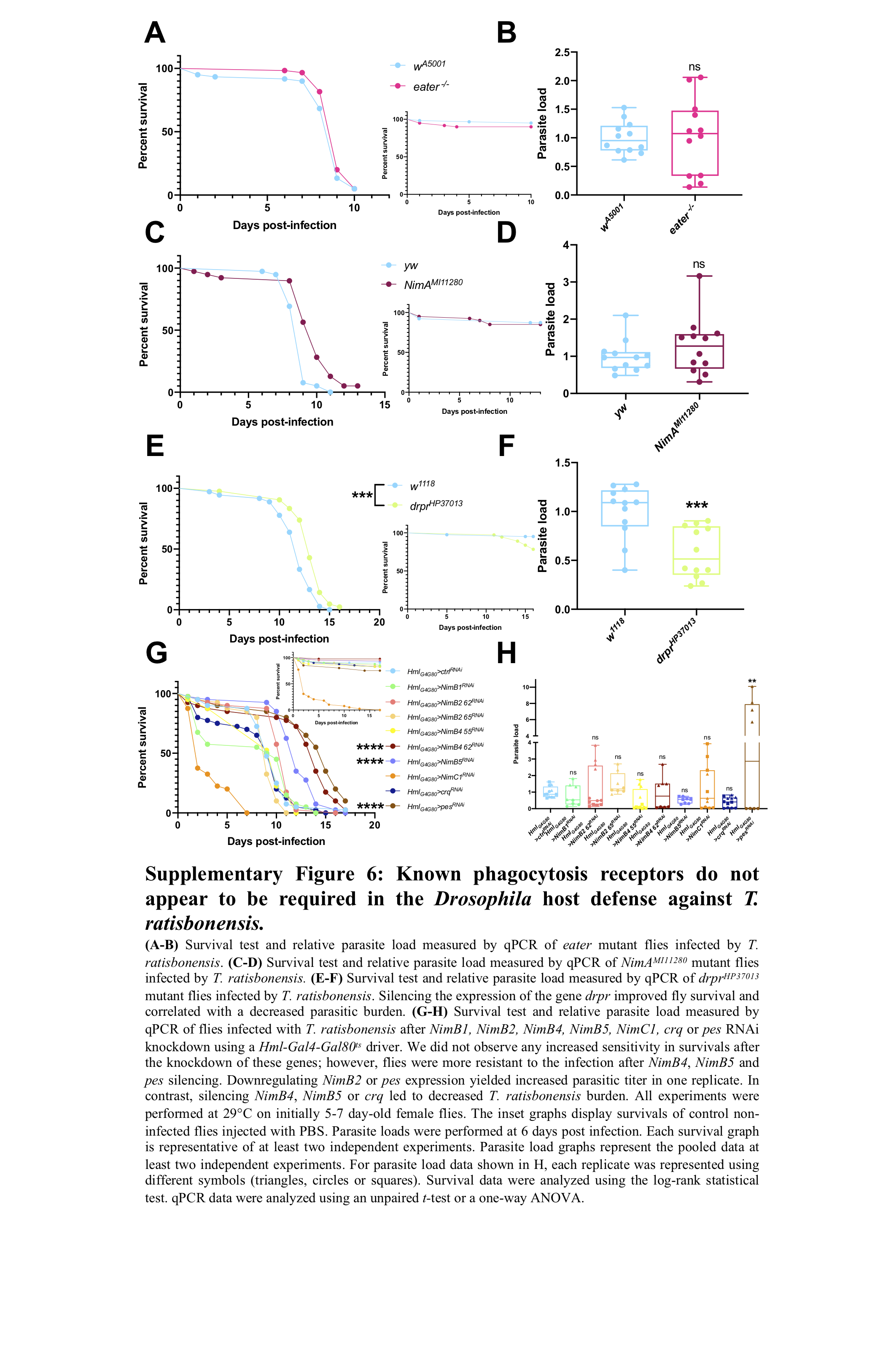

Supplement: Supplementary file 6 [file Image_6.tiff]

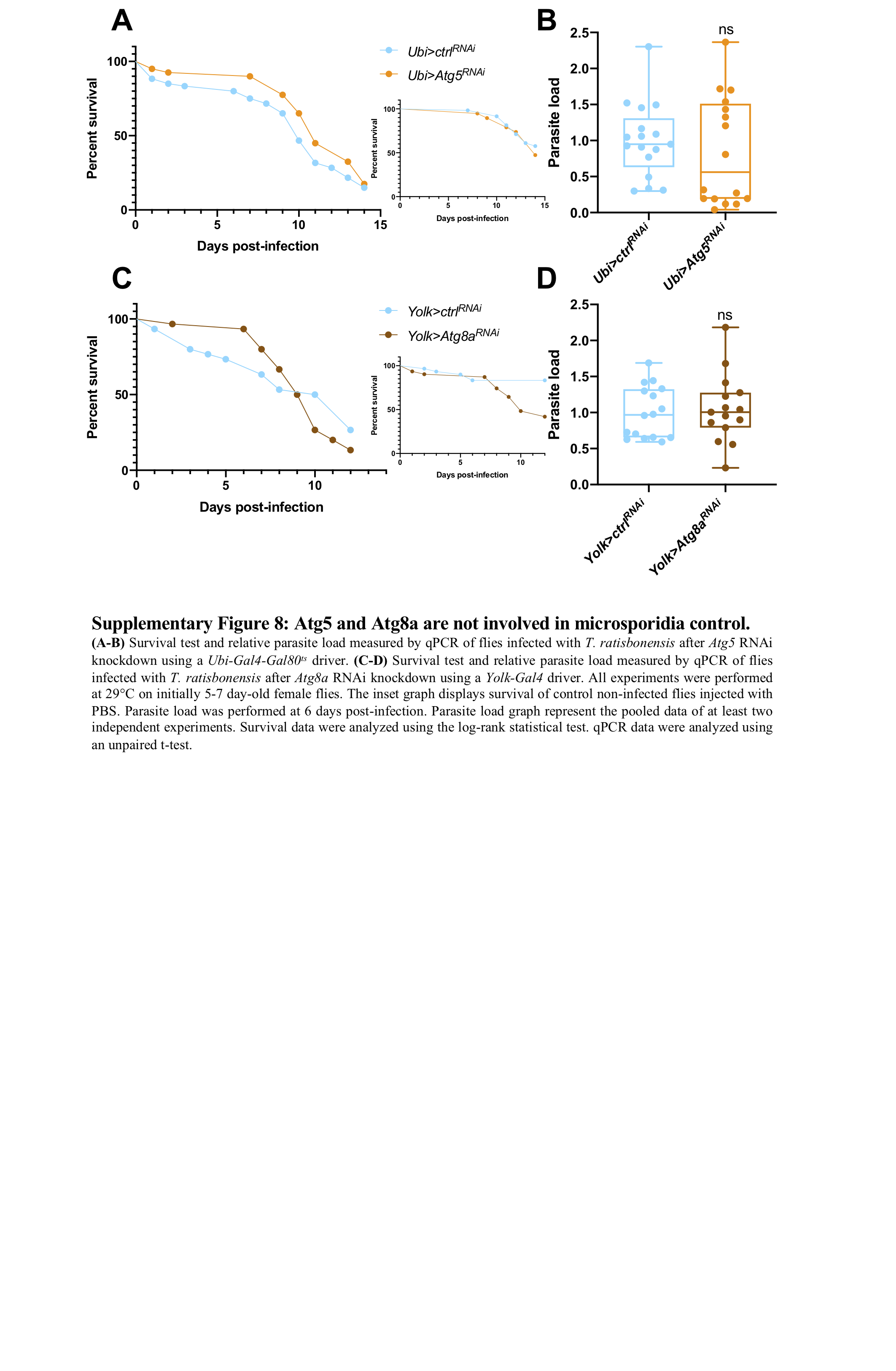

Supplement: Supplementary file 8 [file Image_8.tiff]

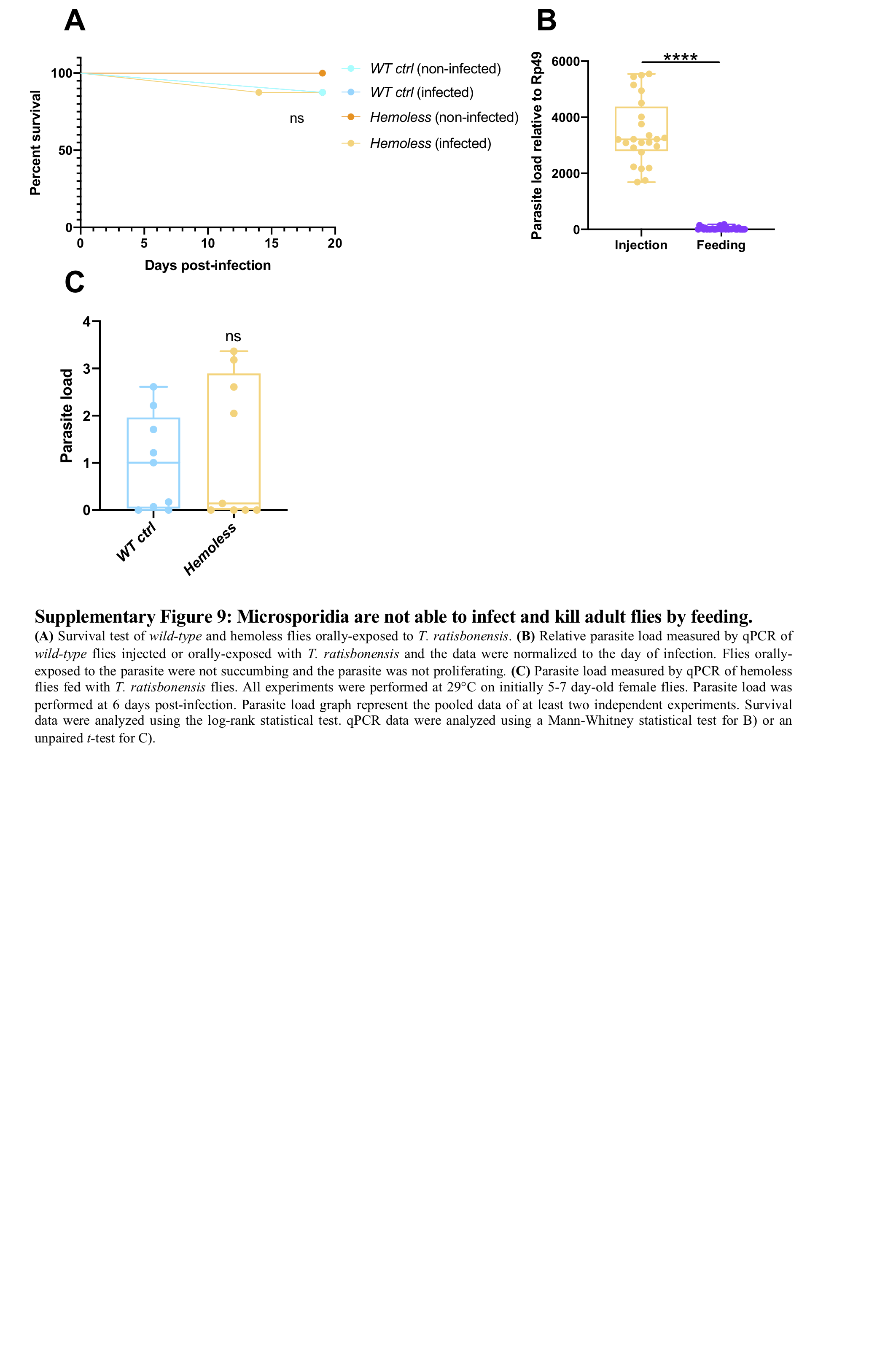

Supplement: Supplementary file 9 [file Image_9.tiff]
